# Impact of manures and fertilizers on yield and soil properties in a rice-wheat cropping system

**DOI:** 10.1371/journal.pone.0292602

**Published:** 2023-11-09

**Authors:** Salwinder Singh Dhaliwal, Vivek Sharma, Vibha Verma, Manmeet Kaur, Prabhjot Singh, Ahmed Gaber, Alison M. Laing, Akbar Hossain

**Affiliations:** 1 Department of Soil Science, Punjab Agricultural University, Ludhiana, India; 2 Department of Chemistry, Punjab Agricultural University, Ludhiana, India; 3 Department of Biology, College of Science, Taif University, Taif, Saudi Arabia; 4 International Maize and Wheat Improvement Center (CIMMYT), Dhaka, Bangladesh; 5 Division of Soil Science, Bangladesh Wheat and Maize Research Institute, Dinajpur, Bangladesh; Directorate of Rapeseed-Mustard Research, INDIA

## Abstract

The use of chemical fertilizers under a rice-wheat cropping system (RWCS) has led to the emergence of micronutrient deficiency and decreased crop productivity. Thus, the experiment was conducted with the aim that the use of organic amendments would sustain productivity and improve the soil nutrient status under RWCS. A three-year experiment was conducted with different organic manures i.e. no manure (M0), farmyard manure @ 15 t ha^-1^ (M1), poultry manure @ 6 t ha^-1^(M2), press mud @ 15 t ha^-1^(M3), rice straw compost @ 6 t ha^-1^(M4) along with different levels of the recommended dose of fertilizer (RDF) i.e. 0% (F1), 75% (F2 and 100% (F3 in a split-plot design with three replications and plot size of 6 m x 1.2 m. Laboratory-based analysis of different soil as well as plant parameters was done using standard methodologies. The use of manures considerably improved the crop yield, macronutrients viz. nitrogen, phosphorus, potassium and micronutrients such as zinc, iron, manganese and copper, uptake in both the crops because of nutrient release from decomposed organic matter. Additionally, the increase in fertilizer dose increased these parameters. The system productivity was maximum recorded under F3M1 (13,052 kg ha^-1^) and results were statistically identical with F3M2 and F3M3. The significant upsurge of macro and micro-nutrients in soil and its correlation with yield outcomes was also observed through the combined use of manures as well as fertilizers. This study concluded that the use of 100% RDF integrated with organic manures, particularly farmyard manure would be a beneficial resource for increased crop yield, soil nutrient status and system productivity in RWCS in different regions of India.

## 1. Introduction

Rice-wheat cropping system (RWCS) is an extremely essential cropping system in India, particularly in Indo–Gangetic plains, (IGP) covering nearly 12.3 Million ha. To fulfil the food requirements of the continuously increasing population, RWCS and cultivation of high-yielding varieties with higher inputs of chemical fertilizers (e.g. synthetic fertilizers and pesticides) have been incorporated into the present agricultural system [1]. Undoubtedly, the approach has evidenced the surplus food grain production [2], however, the use of chemical fertilizers continuously in excessive amounts has resulted in various soil as well as environmental issues such as deficiency of multiple nutrients, soil organic carbon loss, environmental pollution etc which has stagnated the crop yield in the system [3, 4]. In the last decade, stagnated crop productivity has been registered, which may further decline in the coming years due to resource-guzzling practices. Previous studies have suggested the complete organic approach of nutrient management for improving crop yield as well as soil health [5]. However, the sole use of organic nutrient management would result in incomplete organic availability, thus directing the researchers to search for more possible options for nutrient management in crops.

Another approach to tackle this problem is the blending of organic amendments along with conventional fertilizers to replenish soil health and nutrient status. However, the availability of nutrients is lesser in manures and they cannot completely replace all the nutrients required for sustaining crop yield. The combined use of organic manures along with chemical fertilizers has great potential to improve soil quality and productivity of the crop. The application of organic manures such as farmyard manure (FYM), poultry manure (PM), press mud (PrM) and rice straw compost (RSC) in the soil acts as a nutrient reservoir and also helps in recovering soil health. Thus, the inclusion of these amendments in cropping systems could increase on-farm profitability and improve system stability [6]. Various studies have advocated the positive impact of manures and crop residue inclusion on crop yield as well as soil health. In one of the studies, the soil under maize cultivation was treated with sheep manure compost where the maize growth, as well as yield, was found to increase with organic amendments along with improvement in soil aggregation, physical-chemical properties and soil organic matter [7]. In another study, the application of biochar and municipal compost for three years improved the maize yield and resulted in higher Fe uptake along with increased soil organic carbon [8].

Various researchers have reported the beneficial impact of organic amendments on increasing yield outputs of RWCS. Integrated application of chemical fertilizers along with organic amendments such as PrM, RSC, PM and FYM in RWCS increased the system productivity, micronutrient content and uptake by crops [9]. The yield of two different rice varieties was also found to increase through the combined use of chemical fertilizer + PM (50% each) in comparison to the use of chemical fertilizers alone [10]. While previous research has investigated the impact of nutrient management schedules on soil parameters and crop production in various agriculture systems and climatic conditions, but some studies in rice-wheat systems are still lacking. Moreover, the effect of organic manures along with different levels of inorganic fertilizers on system productivity, uptake of nutrients by crops and soil nutrient level has not been studied. Considering the above aspects, a three-year study was conducted to give beneficial and systematic information regarding the effect of organic manures along with inorganic fertilizers on soil quality parameters, system productivity and crop performance including the upsurge of soil macro and micro-nutrients.

## 2. Materials and methods

### 2.1 Experiment details

The trial was conducted in Kharif 2019 at Punjab Agricultural University, Ludhiana, 141004, India (30° 56’ N, 75° 52’ E and 247 m above mean sea level) on sandy loam soil (Typic Ustochrept) with three main plots and five subplot treatments in a split-plot design with plot size 6 m x 1.2 m^2^. In the main plot, nitrogen (N), phosphorus (P) and potassium (K) were applied at 0 (F1), 75 (F2) and 100% (F3) recommended doses of fertilizers (RDF) to rice and wheat crops. The N at 0, 78.7 and 105 kg ha^-1^ and K_2_O@ 0, 22.5 and 30 kg ha^-1^ were applied to rice crop. Whereas N at 0, 93.7 and 125 kg ha^-1^ and P_2_O_5_ @ 0, 46.9 and 62.5 kg ha^-1^ were applied to wheat crop. In the sub-plot, various organic manures i.e., no manure (M0), farmyard manure (FYM) @ 15 t ha^-1^ (M1), poultry manure (PM) @ 6 t ha^-1^ (M2), press mud (PrM) @ 15 t ha^-1^ (M3) and rice straw compost (RSC) @ 6 t ha^-1^ (M4) were mixed in soil before sowing of rice. The composition of nutrients in organic manures used in this work is shown in Table 1.

**Table 1:** Nutrient composition of organic manures.

Organic manures	N	P	K	Zn	Cu	Fe	Mn
	%			mg kg^-1^			
M1	1.01	0.57	1.88	231.2	25.8	1773	221.2
M2	2.35	1.64	2.56	266.6	25.3	3602	220.1
M3	1.42	0.34	1.27	361.7	59.4	1937	319.0
M4	0.83	0.89	2.16	177.3	23.8	1942	463.9

M1: farmyard manure; M2: poultry manure; M3: press mud; M4: rice straw compost

Rice (var. PR 122) was transplanted in *Kharif* season where N (one-third dose) and K (a complete dose) were applied before puddling. The second and third doses of N were given after 7 and 21 days of transplanting. Similarly, during the *Rabi* season, wheat (var. PBW 725) was sown in November. During sowing, N (half dose) and P (full dose) were applied. The remaining N was applied in two split doses at the first and second irrigation.

### 2.2 Preparation and analysis of plant samples

Harvesting of crops was done at maturity where grain as well as straw yields were noted. The grain and straw samples of both rice and wheat were collected at the harvesting stage followed by drying in an oven (65 °C) for 3 days which were then grounded in a willey mill. Nitrogen (N) in the samples was evaluated through Kjeldahl’s method [11]. The concentrations of total nutrients i.e. phosphorus (P), potassium (K), iron (Fe), manganese (Mn), zinc (Zn) and copper (Cu) were achieved through wet-acid digestion of dried samples with a diacid mixture (HNO_3_ and HClO_4_) in 4:1 ratio [12]. Total P in the samples after digestion and filtration was evaluated through the vanado-molybdo-phosphoric-yellow colour method in the nitric acid system [13] on a spectrophotometer, whereas total K was recorded through a flame photometer [14] and the values of total micronutrients were recorded through atomic absorption spectrophotometer (AAS) [15] The concentrations of N, P, K, Zn, Cu, Fe and Mn in manures were also analyzed using the methods described above. The macro as well as micro-nutrient uptakes were calculated using the formula given below.


$$\text{Uptake}\;\left(\text{g}\;{\text{ha}}^{-1}\right)=\frac{\text{Concentration}\left(\text{mg}\;{\text{kg}}^{-1}\right)\times \text{Yield}\left(\text{q}\;{\text{ha}}^{-1}\right)}{10}$$

### 2.3 Cropping system productivity

The productivity of RWCS was recorded by the addition of the total benefits of both crops [16].


Systemproductivity=Wheatyield+Wheatequivalentyieldofrice

Wheat equivalent yield (WEY) for the RWCS was obtained by adding up the obtained wheat yield and WEY of the dry season which was expressed in kg ha^−1^. The WEY was calculated by using the following formula [17]:

$$\text{WEY}={\text{Y}}_{\text{r}}\left({\text{P}}_{\text{r}}/{\text{P}}_{\text{w}}\right)$$

Where, Y_w_ = Wheat seed yield (kg ha^-1^); P_w_ = Wheat seed price (INR per kg seed); P_r_ = Rice grain price (INR per kg grain) and INR = Indian rupee.

The WEY of the RWCS was evaluated by considering INR 19.60 per kg of rice grain and INR 20.15 per kg of wheat seed during the experimental time.

### 2.4 Soil sample analysis

Surface soil samples were collected before the initiation of the experiment and after 3 years following the wheat crop harvest in April 2022. The samples were air-dried and sieved through 2.0 mm plastic sieve. The pH and EC of soil samples were recorded through pH meter and EC meter employing 1:2.5 suspension method [11, 18]. The organic carbon (OC) was analyzed using wet-combustion method [19]. The available N in soil was estimated by the micro-Kjeldahl method [20], whereas available P and K were calculated using 0.5 M NaHCO_3_ extractable P method and ammonium acetate extractable K method using a flame photometer, respectively [21, 22]. Diethylenetriamine-pentaacetic acid (DTPA) extractable soil micronutrients (Zn, Cu, Fe and Mn) were analyzed using DTPA-TEA buffer in the ratio of 1:2, and then their concentrations were determined through AAS [23]. The initial soil properties as determined using the above-mentioned methodologies were: pH, EC and OC = 7.77, 0.22 dS m^-1^ and 0.35%, respectively, N, P and K = 150 .0, 24.40, 133.0 kg ha^-1^, respectively, Zn, Cu, Fe and Mn = 0.48, 0.26, 1.40 and 4.27 mg kg^-1^, respectively.

### 2.5 Statistical analysis

The statistical analysis of experimental data was done through SPSS version 16.0 (SPSS Inc., Chicago, USA) package. Analysis of variance (ANOVA) and Duncan’s multiple range test were used to compare the least significant difference among means at p≤0.05.

## 3. Results

The results regarding the RWCS on the sustainability of crop productivity and build-up of soil nutrients are summarized in the following sections:

### 3.1 Manures and fertilizers effect on crop yield and system productivity

The data of rice and wheat crops for three consecutive years of experiment is given in Table 2. The present study revealed that the additional blending of organic manures along with inorganic fertilizers improved the yield outcomes of both crops. At the end of 3^rd^ year, maximum grain yield in rice was observed in treatment F3M2 (6766 kg ha^-1^) and the results were considerably superior to other treatments (Table 2). Whereas, the highest grain yield in wheat was recorded in treatment F3M1 (6606 kg ha^-1^) which was not statistically different from the results observed in F3M2, F3M3 and F3M4. The system productivity was maximum in F3M2 (13,052 kg ha^-1^), which was not statistically different from treatments F3M1 and F3M3. Thus, the joint use of 100% RDF along with organic manures may prove beneficial for higher rice-wheat productivity.

**Table 2:** Effect of manures and fertilizers on rice yield, wheat yield and system productivity in 3^rd^ year.

Treatments	Rice Yield (kg ha^-1^)			Wheat Yield (kg ha^-1^)			System Productivity of 3^rd^ Year (kg ha^-1^)
	1^st^ Year	2^nd^ Year	3^rd^ Year	1^st^ Year	2^nd^ Year	3^rd^ Year	
F1M0	3920^h^	3808^i^	3752^h^	2640^f^	2684^h^	2702^g^	6352^g^
F1M1	4752^fg^	5167^f^	5225^f^	3952^d^	4197^f^	4336^f^	9419^e^
F1M2	5080^d^	5408^e^	5950^d^	3680^de^	3984^fg^	4149^f^	9938^e^
F1M3	4672^g^	5015^fg^	5495^e^	4096^d^	4200^f^	4275^f^	9620^ef^
F1M4	4928^de^	5104^fg^	5262^f^	3456^e^	3768^g^	4149^f^	9268^f^
F2M0	4680^g^	4744^h^	4852^g^	3888^d^	4128^f^	4552^e^	9272^f^
F2M1	5568^ab^	5656^d^	5908^d^	5696^b^	5992^bc^	6224^b^	11971^c^
F2M2	5592^a^	5888^bc^	6250^c^	5776^b^	5888^c^	5936^c^	12033^c^
F2M3	5544^ab^	5696^cd^	6036^d^	5088^c^	5552^d^	6019^bc^	11891^c^
F2M4	5328^c^	5432^e^	5610^e^	5184^c^	5336^e^	5544^d^	11002^d^
F3M0	4864^ef^	4960^g^	5150^f^	5096^c^	5272^e^	5647^d^	10657^d^
F3M1	5416^bc^	6304^b^	6545^b^	6392^a^	6456^a^	6606^a^	12973^a^
F3M2	5648^a^	5928^a^	6766^a^	6024^ab^	6264^ab^	6470^a^	13052^a^
F3M3	5608^a^	5976^bc^	6473^b^	5648^b^	5912^c^	6489^a^	12787^ab^
F3M4	5544^a^	5864^b^	6116^cd^	6184^a^	6248^ab^	6396^a^	12347^bc^
LSD (0.05)	163	190	210	427	285	215	586

M0: no manure; M1: farmyard manure; M2: poultry manure; M3: press mud; M4: rice straw compost; F1: no fertilizer; F2: 75% recommended dose of fertilizers; F3:100% recommended dose of fertilizers. (Means in a column followed by common lower-case letters are not significantly different).

### 3.2 Manures and fertilizers effect on macronutrient uptake by rice grain and straw

The macronutrient (N, P and K) uptake by rice grain was found to increase considerably with the sole as well as combined use of organic manures and chemical fertilizers. The addition of 100% RDF showed significantly higher results for N uptake in comparison to 75% RDF and no RDF. Among manures, a maximum increase in N uptake was observed with M2 treatment (Table 3).

**Table 3:** Effect of manures and fertilizers on macronutrient uptake (kg ha^-1^) by rice grain and straw.

Treatment	Grain			Straw		
	N	P	K	N	P	K
F1	30.14^c^	14.63^c^	39.77^c^	28.92^c^	14.18^c^	135.90^c^
F2	39.55^b^	18.86^b^	47.60^b^	39.11^b^	17.44^b^	182.54^b^
F3	47.14^a^	21.57^a^	52.58^a^	49.49^a^	20.37^a^	206.93^a^
**LSD (0.05)**	**1.27**	**0.65**	**1.56**	**1.85**	**0.73**	**6.55**
M0	29.83^c^	13.77^c^	36.43^d^	30.23^c^	11.99^d^	110.02^c^
M1	40.44^b^	19.51^ab^	48.98^b^	41.49^ab^	13.50^c^	140.32^b^
M2	44.68^a^	19.99^a^	52.18^a^	42.59^a^	16.34^a^	149.55^b^
M3	40.56^b^	19.64^ab^	49.73^b^	42.16^a^	15.29^b^	157.96^a^
M4	39.20^b^	18.86^b^	45.92^c^	39.39^b^	12.22^d^	142.63^b^
**LSD (0.05)**	**1.64**	**0.83**	**2.02**	**2.38**	**0.94**	**8.46**

M0: no manure; M1: farmyard manure; M2: poultry manure; M3: press mud; M4: rice straw compost; F1: no fertilizer; F2: 75% recommended dose of fertilizers; F3:100% recommended dose of fertilizers. (Means in a column followed by common lower-case letters are not significantly different).

**Fig 1:**
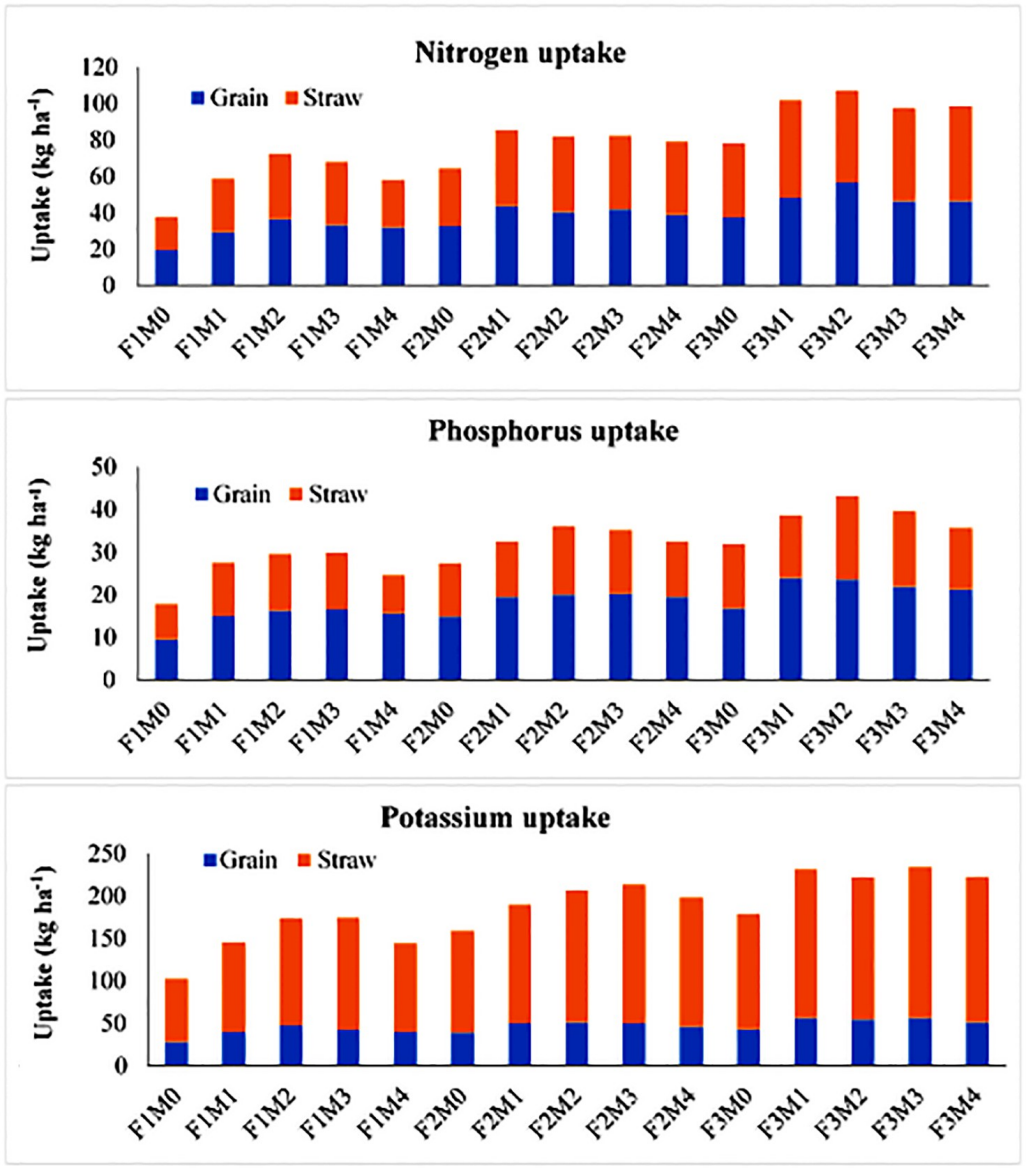
Effect of manures and fertilizers on N, P and K uptake by rice grain and straw. M0: no manure; M1: farmyard manure; M2: poultry manure; M3: press mud; M4: rice straw compost; F1: no fertilizer; F2: 75% recommended dose of fertilizers; F3:100% recommended dose of fertilizers.

The treatments M1, M3 and M4 showed statistically non-significant results although higher than no manure. The interaction data (Fig 1) showed that the uptake of N varied between 19.40–56.80 kg ha^-1^ in all the treatments. Maximum N uptake was observed in F3M2 which was higher than control and other treatments. For P uptake in rice grain, higher results were observed with 75% RDF and single use of manures. Interaction data showed that P uptake in rice grain varied between 9.50–24.03 kg ha^-1^, under different treatments (Fig 1). The highest P uptake was observed in treatment F3M1 and results were statistically identical with F3M2, whereas the minimum value was recorded in F1M0. Whereas for K uptake in rice grain, the addition of 100% RDF recorded significantly higher results in comparison to 75% RDF and no RDF. Whereas, the combined effect of manures and fertilizers resulted in the highest value of K uptake in treatment F3M3 followed by F3M1 and F3M2 (Fig 1).

In the case of rice straw, the addition of 100% RDF showed significantly higher results for N uptake as compared to 75% RDF and no RDF. From manures, the maximum increase in N uptake was recorded with M2 treatment and the results were statistically identical with the treatments M1 and M3 (Table 3). The interaction data showed that the uptake of N varied between 18.40–53.31 kg ha^-1^ in all the treatments (Fig 1) where the maximum N uptake was observed in F3M1 which was significantly at par with the treatments F3M2, F3M3 and F3M4. Whereas the maximum P uptake in rice straw was recorded under M2 which was significantly higher than other organic amendments (M1, M3 and M4) as well as no manure treatment (M0) in the case of different manures. The interaction data showed that P uptake in rice straw ranged from 8.43 to 19.56 kg ha^-1^, under different treatment combinations (Fig 1). The highest P uptake was observed in treatment F3M2 and results were significantly higher than in control as well as other treatments. The K uptake in rice straw was considerably higher with manure M3, which was significantly higher than M0, whereas the results M1, M2 and M4 were statistically identical. From the interactive treatments, the maximum increase (88.54%) was observed in treatment F3M3, which was higher than control and the rest of the treatments (Fig 1).

### 3.3 Manures and fertilizers effect on micronutrient uptake by rice grain and straw

The uptake of Zn, Cu and Mn was found to increase significantly through the application of organic manures along with RDF over the no manure application, whereas Fe uptake was not increased significantly (Table 4).

**Table 4:** Effect of manures and fertilizers on micronutrient uptake (g ha^-1^) by rice grain and straw.

Treatments	Grain				Straw			
	Zn	Cu	Fe	Mn	Zn	Cu	Fe	Mn
F1	157.3^c^	34.8^c^	388.4^c^	92.2^c^	204.4^c^	59.4^c^	2100^c^	256.3^c^
F2	188.2^b^	43.0^b^	470.1^b^	120.8^b^	281.7^b^	75.8^b^	2961^b^	322.2^b^
F3	210.1^a^	51.5^a^	541.7^a^	138.8^a^	353.4^a^	83.8^a^	3362^a^	353.20^a^
**LSD (0.05)**	**5.14**	**2.14**	**13.80**	**3.92**	**11.04**	**3.39**	**135.68**	**14.19**
M0	145.4^d^	31.3^c^	354.8^d^	87.8^c^	213.9^c^	55.0^c^	2208^c^	210.7^c^
M1	194.3^b^	45.1^b^	482.5^b^	122.6^b^	294.7^ab^	78.7^b^	2757^b^	312.8^b^
M2	216.4^a^	49.4^a^	526.9^a^	129.5^a^	307.8^a^	86.0^a^	3088^a^	367.3^a^
M3	187.8^bc^	46.2^b^	511.2^a^	128.9^a^	300.5^ab^	76.5^b^	3093^a^	353.4^a^
M4	182.1^c^	43.5^b^	458.2^c^	117.5^b^	282.2^b^	68.9^c^	2893^b^	308.5^b^
**LSD (0.05)**	**6.64**	**2.77**	**17.82**	**5.06**	**14.24**	**4.38**	**175.20**	**18.32**

M0: no manure; M1: farmyard manure; M2: poultry manure; M3: press mud; M4: rice straw compost; F1: no fertilizer; F2: 75% recommended dose of fertilizers; F3:100% recommended dose of fertilizers. (Means in a column followed by common lower-case letters are not significantly different).

**Fig 2:**
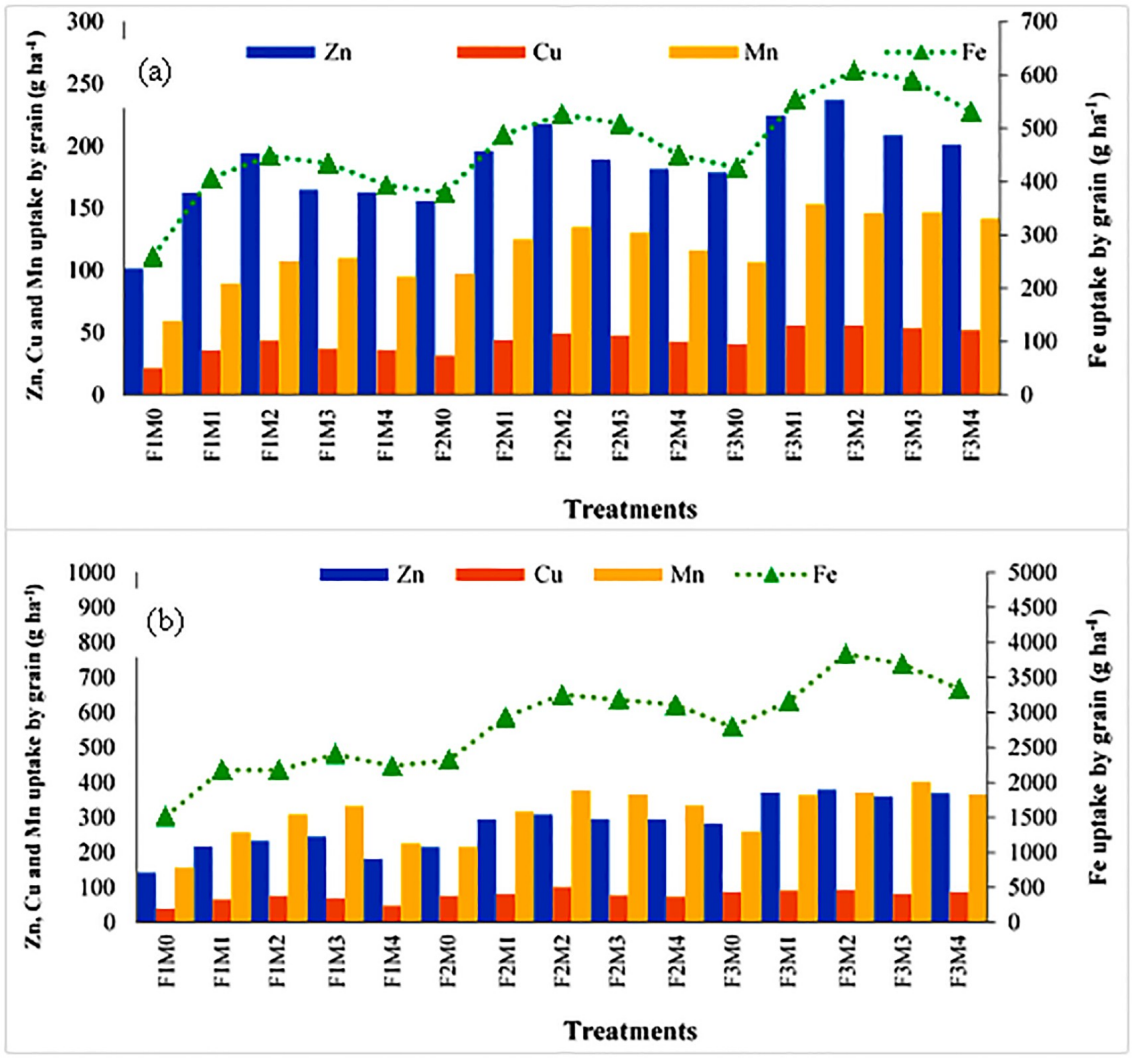
Effect of manures and fertilizers on micronutrient uptake by (a) rice grain and (b) straw. M0: no manure; M1: farmyard manure; M2: poultry manure; M3: press mud; M4: rice straw compost; F1: no fertilizer; F2: 75% recommended dose of fertilizers; F3:100% recommended dose of fertilizers.

Micronutrient (Zn, Cu, Fe and Mn) uptake among all the treatments ranged from 101.3–237.0, 21.57–55.86, 259.75–607.47 and 59.13–153.15 g ha^-1^, respectively. Among manures, Zn uptake was significantly higher under M2 (45.50%) than M0 as well as from other treatments. A similar trend was seen for Cu, Fe and Mn uptake. In the case of Fe and Mn, the results of M3 were statistically non-significant with M2. The addition of 100% RDF recorded significantly higher results for micronutrients (Zn, Cu, Fe and Mn) than 75% RDF and without RDF. The interactive data of different treatments demonstrated that the treatment F3M2 showed maximum Zn uptake (237.0 g ha^-1^). The Cu uptake was maximum recorded under F3M1 (55.86 g ha^-1^) which was not statistically different from treatments F3M2 and F3M4 and a similar trend was recorded for Mn uptake (Fig 2a).

Uptake of Zn, Cu and Mn in rice straw enhanced significantly through the use of organic manures along with RDF over the no manure application, whereas Fe uptake was not significantly increased. The Zn, Cu, Fe and Mn uptake varied between 143.71–380.93, 39.64–92.80, 1514–3832 and 155.88–403.28 g ha^-1^, respectively, under various treatments. Among manures, Zn uptake was significantly higher under M2 (72.40%) than M0, however, it was statistically identical with M1 and M3. A similar trend was observed for uptake of Cu, Fe and Mn. Also, the addition of 100% RDF recorded significantly higher results for micronutrients (Zn, Cu, Fe and Mn) than 75% RDF and without RDF. The interactive data of different treatments demonstrated that the treatment F3M2 showed maximum Zn uptake (380.93 g ha^-1^) (Fig 2b). The Cu uptake was also observed to be maximum under F3M2 (92.80 g ha^-1^), which was not statistically different from treatments F3M1 and F3M4. Likewise in Mn, F3M2 showed maximum uptake (403.28 g ha^-1^), which was statistically identical to F3M3.

### 3.4 Manures and fertilizers effect on macronutrient uptake by wheat grain and straw

The overall N, P and K uptake in wheat grain ranged from 14.17–47.31 kg ha^-1^, 8.52–22.52 kg ha^-1^ and 13.52–45.42 kg ha^-1^ in all the treatments (Table 5).

**Table 5:** Effect of manures and fertilizers on macronutrient uptake (kg ha^-1^) by wheat grain and straw.

Treatments	Grain			Straw		
	N	P	K	N	P	K
F1	23.90^c^	12.54^c^	23.11^c^	20.68^b^	11.23^c^	76.26^c^
F2	37.50^b^	19.06^b^	34.39^b^	34.34^a^	17.43^b^	120.39^b^
F3	43.29^a^	21.33^a^	41.03^a^	43.68^a^	21.36^a^	146.30^a^
**LSD (0.05)**	**1.28**	**0.67**	**1.34**	**1.76**	**0.89**	**6.82**
M0	26.76^c^	14.03^c^	24.06^c^	26.53^b^	12.77^d^	88.94^b^
M1	37.27^a^	18.76^a^	36.22^a^	39.24^a^	18.02^b^	123.99^a^
M2	37.98^a^	19.18^a^	32.65^b^	31.77^a^	19.69^a^	118.69^a^
M3	37.31^a^	18.88^a^	34.89^a^	34.07^a^	17.05^b^	122.42^a^
M4	35.15^b^	17.35^b^	36.42^a^	32.91^a^	15.86^c^	117.28^a^
**LSD (0.05)**	**1.65**	**0.86**	**1.73**	**2.27**	**1.14**	**8.80**

M0: no manure; M1: farmyard manure; M2: poultry manure; M3: press mud; M4: rice straw compost; F1: no fertilizer; F2: 75% recommended dose of fertilizers; F3:100% recommended dose of fertilizers. (Means in a column followed by common lower-case letters are not significantly different).

**Fig 3:**
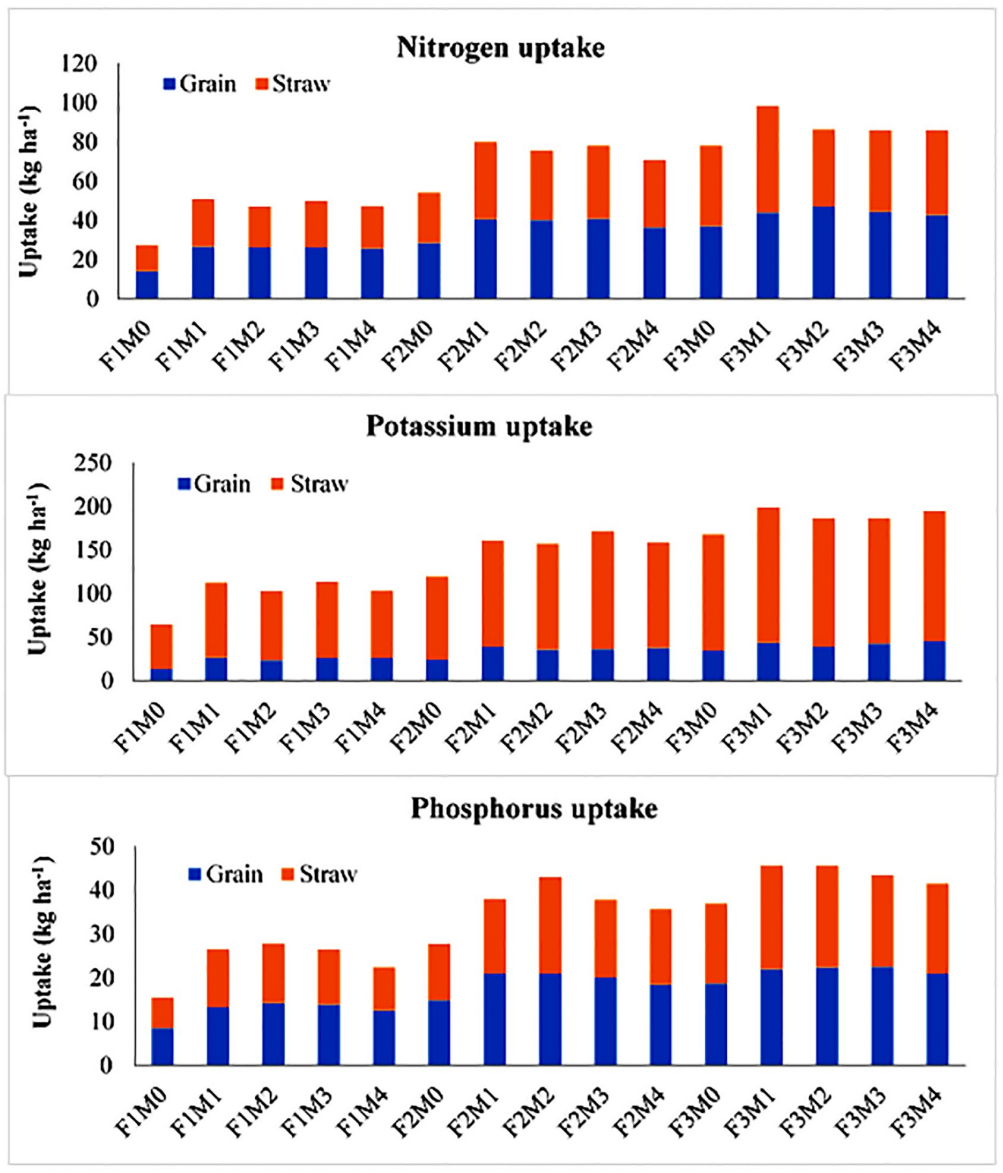
Effect of manures and fertilizers on N, P and K uptake by wheat grain and straw. M0: no manure; M1: farmyard manure; M2: poultry manure; M3: press mud; M4: rice straw compost; F1: no fertilizer; F2: 75% recommended dose of fertilizers; F3:100% recommended dose of fertilizers.

Comparing different manures, maximum N uptake was observed in PM (M2) treatment which was higher as compared to no manure treatment i.e. M0 (11.72%) as well as other amendments. For P uptake also, the maximum value was recorded under M2 which was statistically at par with M1 and M3. However, K uptake was recorded highest under M4 treatment which was statistically identical to treatments M1 and M3. On the other hand, the level of RDF application had a significant impact on micronutrient uptake. The interaction data showed that a combination of various treatments had a considerable effect on N and K uptake, however non-significant variation was recorded in P uptake (Fig 3). Maximum N uptake was recorded under F3M2, whereas K uptake was maximum under F2M4, which was statistically identical with F3M1 and F3M4.

The uptake of N, P and K in wheat straw varied between 13.31–54.36 kg ha^-1^, 7.03–23.65 kg ha^-1^ and 51.21–155.32 kg ha^-1^ under various treatments (Table 5). Interactive data showed non-significant variation for K uptake. All the treatments involving the use of manures considerably enhanced the N uptake as compared to no manure treatment (M0) and a similar trend was seen for K uptake. Also, the RDF application at 75% RDF and 100% RDF showed statistically identical results for N uptake but significantly higher than no RDF application. However, for P and K uptake the results of F3 were significantly higher than F1 and F2. The data pertaining to N and P uptake demonstrated the highest uptake under F3M1 which was statistically identical to F3M2 in the case of P uptake (Fig 3). All the other treatments also recorded significantly higher results than the control treatment.

### 3.5 Manures and fertilizers effect on micronutrient uptake by wheat grain and straw

A significant effect of organic manures on micronutrient uptake was observed in rice. Uptake of Zn, Cu, Fe and Mn in wheat grain varied between 65.86, 5.78, 218.54 and 42.35 g ha^-1^ to 202.94, 21.80, 765.03 and 178.75 g ha^–1^, respectively, under various treatments. The residual effect of FYM showed significant results for all the micronutrient uptake in comparison to other manures. The level of RDF application also affected the micronutrient uptake. The results of 100% RDF were significantly higher than 75% RDF and no RDF (Table 6).

**Table 6:** Effect of manures and fertilizers on micronutrient uptake (g ha^-1^) by wheat grain and straw.

Treatment	Grain				Straw			
	Zn	Cu	Fe	Mn	Zn	Cu	Fe	Mn
F1	104.2^c^	9.90^c^	335.7^c^	84.6^c^	108.6^c^	28.69^c^	2164^c^	86.96^c^
F2	162.5^b^	15.48^b^	542.6^b^	138.0^b^	181.4^b^	53.56^b^	3643^b^	146.8^b^
F3	187.0^a^	19.99^a^	671.3^a^	162.4^a^	220.6^a^	73.68^a^	5196^a^	189.1^a^
**LSD (0.05)**	**4.73**	**0.70**	**31.18**	**4.93**	**9.59**	**2.85**	**242.80**	**13.94**
M0	116.7^d^	11.35^c^	392.4^c^	91.8^c^	132.2^c^	40.09^c^	2714^c^	101.8^b^
M1	171.2^a^	16.45^a^	592.1^a^	144.5^a^	177.0^b^	58.62^a^	4047^a^	152.8^a^
M2	161.1^b^	16.09^ab^	584.6^a^	133.8^b^	165.6^b^	52.53^b^	3653^b^	152.0^a^
M3	158.3^b^	16.49^a^	509.5^b^	132.9^b^	202.2^a^	57.50^a^	4215^a^	151.3^a^
M4	148.7^c^	15.25^b^	503.9^b^	138.8^ab^	174.1^b^	51.13^b^	3716^b^	147.1^a^
**LSD (0.05)**	**6.11**	**0.90**	**40.24**	**6.36**	**12.38**	**3.60**	**313.44**	**17.99**

M0: no manure; M1: farmyard manure; M2: poultry manure; M3: press mud; M4: rice straw compost; F1: no fertilizer; F2: 75% recommended dose of fertilizers; F3:100% recommended dose of fertilizers. (Means in a column followed by common lower-case letters are not significantly different).

**Fig 4:**
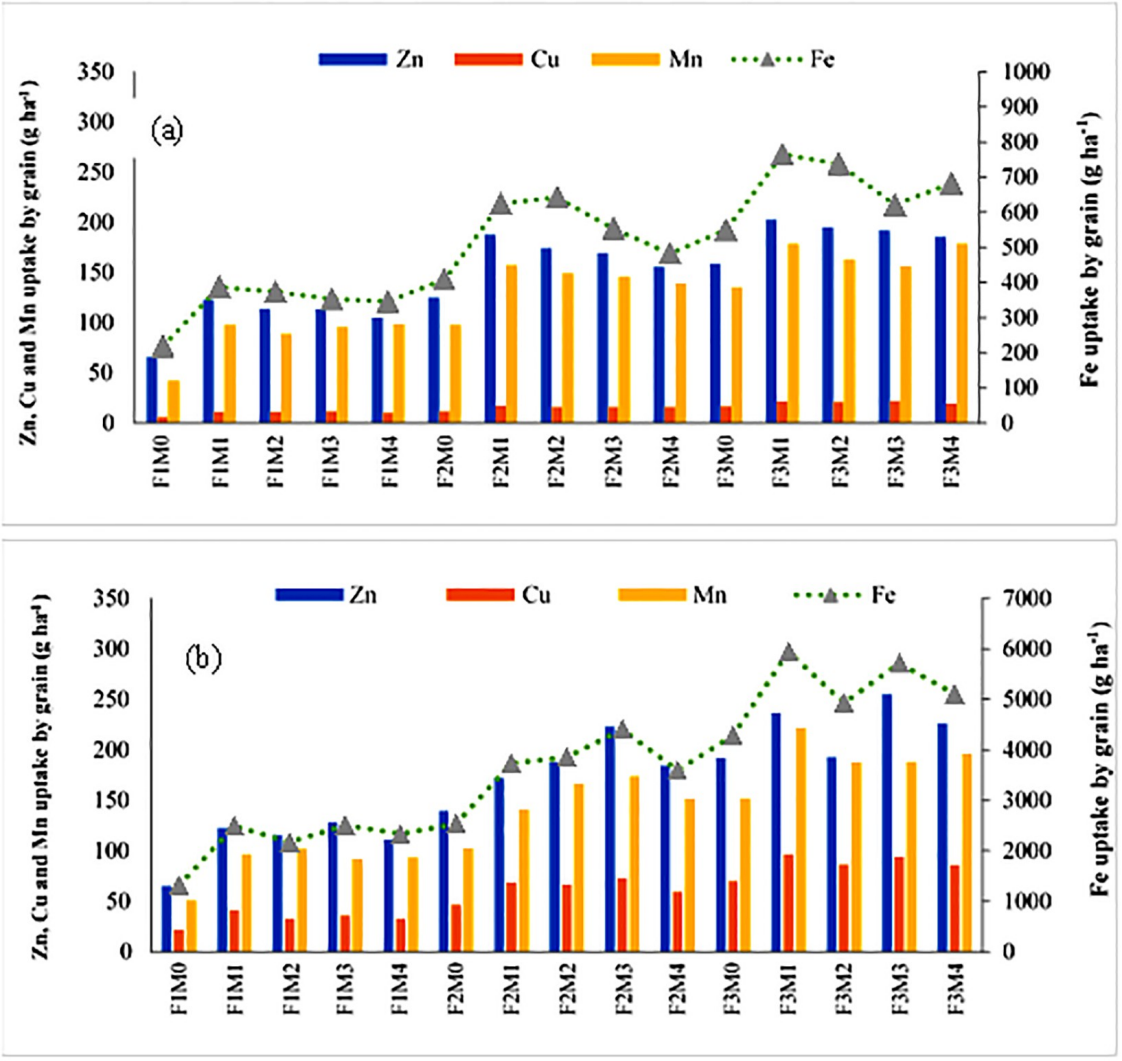
Effect of manures and fertilizers on micronutrient uptake by wheat (a) grain and (b) straw. M0: no manure; M1: farmyard manure; M2: poultry manure; M3: press mud; M4: rice straw compost; F1: no fertilizer; F2: 75% recommended dose of fertilizers; F3:100% recommended dose of fertilizers.

The uptake of Zn, Cu, Fe and Mn, in wheat straw varied between 65.47–254.88, 21.39–96.92, 1313–5944 and 50.71–221.47 g ha^-1^ under various treatments. Among manures, the highest Zn and Fe uptake was recorded with the residual effect of M1, whereas Cu and Mn uptake was highest with M2. The Zn uptake did not differ significantly among other manures. The residual effect of M3 and M1 was statistically at par for Fe and Cu uptake. Interactive data of different treatments in wheat grain demonstrated that the treatment F3M1 showed maximum Zn uptake (202.94 g ha^-1^), which was considerably higher than the control and all other treatments (Fig 4a). The Cu uptake was recorded as a maximum under F3M3 (21.80 g ha^-1^) whereas Mn uptake was maximum in treatment F3M4 (178.80 g ha^-1^) which was statistically identical to F3M1. Similarly, Zn and Cu uptake in wheat straw were observed to be maximum in treatments F3M3 (254.88 g ha^-1^) and F3M1 (96.92 g ha^-1^), respectively (Fig 4b).

### 3.6 Effect on soil properties and macronutrients buildup

The impact of organic amendments on soil properties is given in Tables 7 and 8. The use of organic manures (M1, M2 and M3) favoured the acidification of soil pH as confirmed by slightly lower pH values in comparison to unamended soil (M0) (Table 7). The addition of RDF also slightly reduced the soil pH in comparison to the results when no RDF was added to the soil.

**Table 7:** Effect of manures and fertilizers on soil properties.

Treatments	pH	EC	OC	N	P	K	Zn	Cu	Fe	Mn
		dS m^-1^	(%)	(kg ha^-1^)			(mg kg^-1^)			
F1	7.71	0.24	0.37^c^	216.2^c^	30.89^c^	181.5^c^	2.15^c^	0.98^c^	22.35^c^	6.04^b^
F2	7.76	0.26	0.41^b^	246.8^b^	36.87^b^	211.8^b^	2.30^b^	1.04^b^	23.72^b^	6.47^a^
F3	7.77	0.26	0.43^a^	291.7^a^	41.43^a^	224.0^a^	2.36^a^	1.14^a^	25.45^a^	6.74^a^
**LSD (0.05)**	NS	NS	0.01	8.67	1.59	7.59	0.03	0.02	0.42	0.31
M0	7.70	0.22^b^	0.33^c^	224.3^d^	30.37^d^	191.6^d^	1.72^d^	0.96^c^	19.76^d^	5.37^c^
M1	7.67	0.28^a^	0.42^b^	266.6^b^	37.27^b^	224.4^a^	2.22^c^	1.15^a^	25.21^b^	7.34^a^
M2	7.80	0.24^b^	0.40^b^	285.3^a^	42.74^a^	204.4^bc^	2.35^b^	1.13^a^	26.14^a^	6.77^b^
M3	7.75	0.28^a^	0.46^a^	237.1^c^	35.78^c^	199.0^cd^	2.68^a^	0.94^c^	24.30^c^	5.53^c^
M4	7.82	0.24^b^	0.41^b^	244.6^c^	35.80^bc^	209.7^b^	2.38^b^	1.07^b^	23.79^d^	7.07^ab^
**LSD (0.05)**	NS	0.02	0.02	11.19	2.05	9.80	0.04	0.03	0.55	0.40

M0: no manure; M1: farmyard manure; M2: poultry manure; M3: press mud; M4: rice straw compost; F1: no fertilizer; F2: 75% recommended dose of fertilizers; F3:100% recommended dose of fertilizers. (Means in a column followed by common lower-case letters are not significantly different).

**Table 8:** Interactive effect of manures and fertilizers on soil properties.

Treatments	pH	EC	OC	N	P	K	Zn	Cu	Fe	Mn
		dS m^-1^	(%)	(kg ha^-1^)			(mg kg^-1^)			
F1M0	7.77	0.20^e^	0.31^f^	198.0^h^	24.75^g^	161.5^f^	1.50^h^	0.84^h^	19.43^g^	4.22^e^
F1M1	7.77	0.26^bc^	0.41^d^	233.9^fg^	31.19^e^	205.7^c^	2.13^f^	1.09^d^	22.95^ef^	6.78^bc^
F1M2	7.76	0.22^de^	0.39^d^	246.0^ef^	39.35^cd^	180.3^ef^	2.32^cd^	1.08^e^	24.62^d^	6.76^bc^
F1M3	7.82	0.26^bc^	0.40^d^	203.0^h^	32.48^e^	183.9^e^	2.46^b^	0.89^g^	22.64^f^	5.37^d^
F1M4	7.88	0.24^cd^	0.35^e^	200.0^h^	26.67^f^	176.3^ef^	2.31^d^	0.98^f^	22.12^f^	7.04^b^
F2M0	7.78	0.22^de^	0.34^ef^	215.1^gh^	29.92^ef^	199.7^de^	1.83^g^	0.90^g^	19.67^g^	5.75^d^
F2M1	7.63	0.28^ab^	0.42^cd^	259.4^de^	37.56^d^	225.6^abc^	2.22^e^	1.14^bcd^	24.66^cd^	7.29^ab^
F2M2	7.76	0.24^cd^	0.40^d^	279.3^d^	42.63^bc^	213.7^bcd^	2.33^cd^	1.13^bcde^	26.44^b^	6.69^bc^
F2M3	7.71	0.28^ab^	0.48^ab^	234.6^fg^	36.13^d^	201.8^cd^	2.75^a^	0.92^g^	24.11^d^	5.55^d^
F2M4	7.84	0.24^cd^	0.42^c^	246.6^ef^	38.09^d^	221.4^abc^	2.39^bcd^	1.10^cde^	23.71^de^	7.08^b^
F3M0	7.86	0.24^cd^	0.35^e^	259.7^de^	36.45^d^	213.5^bc^	1.84^g^	1.15^bc^	20.17^g^	6.13^cd^
F3M1	7.74	0.28^ab^	0.44^cd^	307.5^b^	43.06^b^	241.8^a^	2.31^d^	1.24^a^	28.01^a^	7.97^a^
F3M2	7.78	0.24^cd^	0.41^d^	330.6^a^	46.23^a^	219.1^bcd^	2.40^bc^	1.17^b^	27.35^a^	6.86^b^
F3M3	7.73	0.30^a^	0.51^a^	273.6^d^	38.75^d^	214.4^bc^	2.83^a^	1.01^f^	26.17^b^	5.66^d^
F3M4	7.84	0.28^ab^	0.45^bc^	287.2^c^	42.63^b^	231.4^ab^	2.44^b^	1.15^bc^	25.55^bc^	7.09^b^
LSD (0.05)	NS	0.02	0.03	11.19	3.56	19.95	0.08	0.05	0.95	0.70

M0: no manure; M1: farmyard manure; M2: poultry manure; M3: press mud; M4: rice straw compost; F1: no fertilizer; F2: 75% recommended dose of fertilizers; F3:100% recommended dose of fertilizers. (Means in a column followed by common lower-case letters are not significantly different).

The interaction table showed non-significant variation in soil pH on the combined treatment of organic manures and fertilizers. The addition of M1 and M3 showed significantly higher results for EC, whereas M2 showed results that were significantly at par with control. Also, the addition of RDF did not considerably affect the soil EC. The interaction table predicted the highest EC value with treatment F3M3 (0.30 dS m^-1^) and the results were not statistically different from treatments F2M1, F3M1, F1M3 and F1M4. Whereas, the maximum value of OC was observed in treatment F3M3 (0.51%). The impact of increasing the inorganic fertilizer dose was non-significant, whereas the addition of manures significantly affected the OC. Maximum OC was obtained with M3 which was significantly higher than other organic amendments and the least was recorded in no manure addition.

The soil macronutrient data showed that the available N content varied significantly between 198.0–330.6 kg ha^−1^ with the combined use of fertilizers as well as manures (Table 8). The addition of PM showed the maximum increase in N (27.23%) in comparison to control and the results were significantly higher than the addition of other organic fertilizer sources. Likewise, the results for 100% RDF were significantly more as compared to the addition of 75% RDF and no RDF. Within the treatments, PM application with 100% RDF showed the highest available N content, which was 66.66% higher than the control. Whereas, available P varied between 24.75 kg ha^−1^ (F1M0)–46.23 (F3M2) kg ha^−1^. Among the different manures, PM resulted in higher available P content, and it was significantly higher than other organic amendments. In the case of RDF, 100% addition showed significantly higher results than 75% RDF and no RDF. The available K content varied between 161.50–241.77 kg ha^−1^ in soil and was minimal in control. The interaction table showed that the highest K was recorded under F3M1, which was 49.68% higher than F1M0. The results of treatment F3M1 were not statistically different from F2M1, F2M4 and F3M4.

### 3.7 Effect on micronutrient build-up in soil

The DTPA-extractable Zn in various treatments ranged between 1.51–2.83 mg kg^−1^ (Tables 7 and 8). The maximum Zn was observed in F3M3 i.e. 100% RDF + PrM, which was statistically similar to 75% RDF + PrM and the least value for Zn was observed in F1M0. Data also demonstrated that the Zn content with 100% RDF was significantly higher as compared to the treatments with 75% RDF irrespective of the manures used. The DTPA-extractable Cu varied between 0.84–1.24 mg kg^−1^ in various treatments. The maximum value for Cu content was observed in treatment involving 100% RDF + FYM, which was significantly higher than other treatments. Thus, the use of FYM showed the highest results for Cu content. For DTPA-extractable Fe, values ranged between 19.43–28.01 mg kg^−1^ in various treatments and the highest value was observed in treatment F3M1 i.e., 100% RDF + FYM, which was 44.15% higher than the control. For DTPA-extractable Mn, the content varied between 4.22 (F1M0)–7.97 (F3M1) mg kg^−1^ and the highest value was observed through the use of 100% RDF and organic manures, which was statistically at par with the treatments involving 75% RDF. Thus, FYM and RSC showed significantly higher Mn content over the control.

### 3.8 Correlation between yield and soil properties

**Fig 5:**
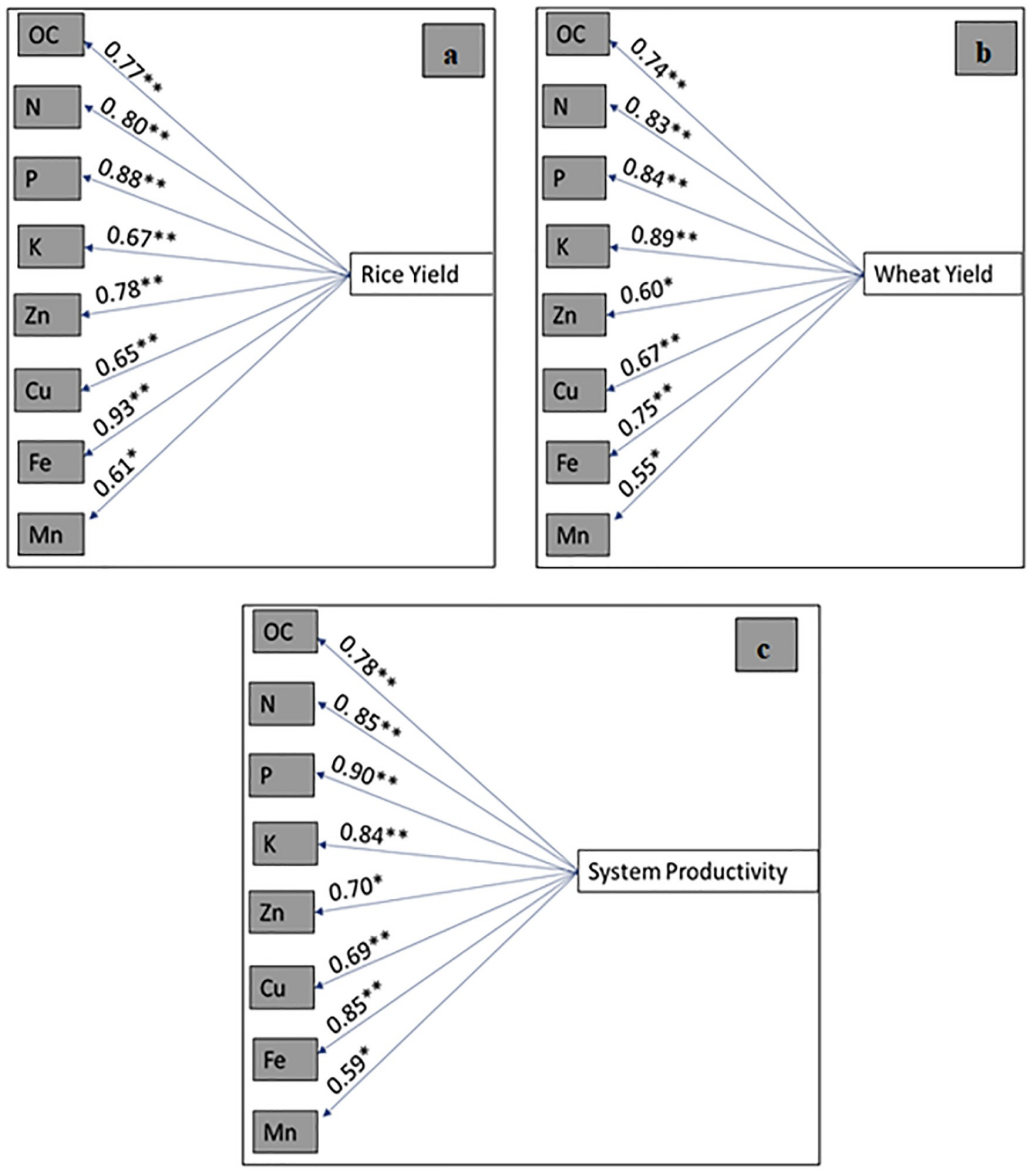
Correlation between yield, soil properties and nutrient content: (a) shows the significant positive correlation between rice yield and soil properties; (b) shows the significant positive correlation between wheat yield and soil properties; and (c) shows the correlation between system productivity and soil properties.

The correlation between crop yields (rice yield, wheat yield and system productivity) and soil properties is given in Fig 5(a) & 5(b). The data in Fig 5(a) shows that the yield of both crops and system productivity possessed a positive and significant correlation with all soil properties except pH and EC. The maximum significance and positive correlation of rice yield was with DTPA-extractable Fe with a correlation coefficient of 0.93, demonstrating that the contribution of soil Fe content was maximum towards rice yield. On the other hand, wheat yield showed the highest positive correlation with the available K value (Fig 5(b)). In the case of system productivity, a maximum significant correlation was found with available P content (Fig 5(c)).

## 4. Discussion

### 4.1 Manures and fertilizers effect on yield sustainability of rice and wheat

The present results showed an increase in yield for successive years except for control in rice, in which there was a depletion of soil nutrient status as no nutrients were added through the exogenous supply. The organic amendments contributed to the soil fertility status by supplying nutrients through the decomposed organic matter. Also, the release of different organic acids from organic matter favour the solubilization of native soil nutrients and increases their availability to plants [24]. After 3^rd^ year, the maximum yield of rice recorded under F3M2 was because of the higher nutrient status of PM in comparison to the other manures and RSC [24]. Poultry manure also possesses a high rate of mineralization as compared to other organic sources; thus the early release of nutrients makes them readily available to the succeeding crop [25]. Moreover, a full dose of RDF was applied under F3M2 as compared to F2M2 under which 75% RDF was applied, which resulted in higher availability of nutrients [26, 27]. On the other hand, the maximum residual effect of FYM on wheat crop yield was recorded because of the slow rate of mineralization of FYM [28, 29]. Also, the higher yield under F3 as compared to F2 in wheat, irrespective of manures used, was attributed to the higher availability of nutrients [26, 27].

### 4.2 Manures and fertilizers effect on macronutrient uptake in rice and wheat grains

The increase in macronutrient uptake in grain and straw of rice might be due to organic matter decomposition which resulted in the assimilation of macronutrients into the soil matrix, thus making soil a nutrient reservoir [30]. Biofertilizers play a key role in N fixation, solubilization of P, and mobilization of more amounts of nutrients in a readily available form in the soil. Further, these released nutrients become easily available for plant uptake. Additionally, the improved grain and straw yield along with the presence of a sufficient amount of N in organic manures and inorganic fertilizers led to the improved uptake of macronutrients. Uptake of N by plant is a good indicator of a crop’s N utilization efficiency. In the present study, when PM was applied along with a 75% NPK, N absorption enhanced due to a higher amount of nutrients from PM. Similar results were recorded by Mia et al. [31] where N and K uptake was found to increase with enhancing levels of PM application. Whereas, the P and K uptake were found to be the maximum in treatments involving FYM and inorganic fertilizers. The positive effect of FYM on K uptake was due to the fact that in addition to FYM acting as a source of K, it also influenced the availability of native and applied K by releasing organic colloids with higher cation exchange sites to attract K from non-exchangeable pool. Additionally, the applied K favoured crop growth and higher accumulation of biomass through increased K uptake. Similar results were observed for P uptake. The above results were corroborated with the work conducted by Majumdar et al. [32].

In wheat grain, the highest N uptake was noted in treatment involving PM; whereas the maximum N uptake for wheat straw was found in treatment involving FYM. When FYM was applied with a 75% NPK fertilizer, N absorption was found to increase in the case of wheat straw. The FYM has a higher C:N ratio, which resulted in the immobilisation of N [33], while the sole application of inorganic fertilizers led to N loss because of leaching as well as volatilization [34, 35]. Farmyard manure improved soil properties, thus resulting in higher root density and reduced leaching, which improved the nutrient absorption capability of the crop. Thus, using organic and inorganic sources in combination improved the micronutrient absorption in wheat straw [36]. Whereas, in the case of wheat grain residual effect of rice might have resulted in lesser uptake of N from the soil thus leading to lesser accumulation of N in treatment involving FYM. Moreover, soil experiments showed a higher amount of N in PM which further append to the higher N uptake in treatment involving PM. In the case of K, treatment F2M4 involving RSC along with inorganic fertilizers exhibited maximum K uptake. This may be due to the increase in the soil Fe^2+^ and Mn^2+^, caused by RSC, which release K from exchange complexes. These outcomes were similar to those obtained by Kaur and Benipal [37] in which recycling of rice straw to the field improved available K in soil to a considerable level more than manure. Also, the maximum level of K was observed in RSC in the present study which resulted in increased K uptake in treatment involving RSC.

### 4.3 Manures and fertilizers effect on micronutrient uptake in rice and wheat grains

The higher micronutrient uptake through combined use of organic manures as well as inorganic fertilizers might be because of increased micronutrient bioavailability in soil, which improved their uptake in rice as compared to control [38]. Poultry manure is an excellent animal manure which helps to boost the productivity of soil as compared to other manures and provides significant improvement in organic C, available P and exchangeable cations [39]. Also, organic manures possess positive effects on plant growth not only because of the temporal availability of essential minerals but also because of enhancement in soil physical, chemical and biological properties which help in increasing the uptake of micronutrients in grain and straw of rice. Additionally, the uptake of micronutrients was also related to the interaction with other nutrients as a higher concentration of N helps in improving the number of Zn transporters and Zn chelating nitrogenous substances [40–42]. Manganese also shows a favourable interaction with N as nitrate possesses the ability to promote Mn availability and Mn uptake in rice [43, 44].

In the present study, soil organic matter forms a number of complex biochemical reactions after the application of manures in the soil. The presence of carboxylic and phenolic functional groups in soil organic matter upon dissociation enhanced the negative charge on soil and also improved its resistance to acidification. Also, stabilization of soil structure was achieved through organic fertilizers which reduced the N loss and improved N levels in soil. More N fertilization improved the Zn and Fe uptake and translocation in wheat roots and shoots [41], resulting in increased Zn and Fe uptake in wheat grain and straw [42]. On the other hand, Cu and Mn uptake was maximum in treatment involving PM along with inorganic fertilizers. The application of PM provides excess levels of organic matter affecting mineral solubility as well as plant availability. This also stimulates the activity of microorganisms which temporarily decreases the nutrient availability while improving the solubility of other nutrients during the breakdown of organic matter in soil. So, PM increased the available quantities of Cu and Mn in soil which were easily taken up by wheat thus enhancing their uptake in crop.

### 4.4 Manures and fertilizers effect on the build-up of soil chemical properties

The lower pH of organically amended soil as compared to unamended soils might be because of organic acids released from microbial decomposition of the incorporated manures, which lowered the soil pH below their initial values. Adekiya et al. [24] reported similar results with organic amendments to soils. The application of NPK fertilizer also accelerated soil acidification because of the leaching of bases from the surface of the soil. The incorporation of N fertilizers led to the displacement of acidic ions and basic cations from the sorption complex, which increased the active acidity and the leaching of base cations with accompanying anions, respectively. Also, the NH_4_^+^ absorption by plants/microorganisms along with secretion of H^+^ during nitrification, contribute to intensifying soil acidification [45]. The increase in EC with manure application was associated with the increase in soluble salt concentration sourced from feed additives and the solubilization of native minerals due to a lowering in soil pH. The acids released from manure decomposition undergo chemical reactions with soluble salts present in the soil which get converted to either soluble salts or experience increased solubility. The increase in OC was due to the addition of organic matter through manures. The higher OC in the case of PrM addition was because of a higher level of PrM added to soil as compared to other manures.

Similarly, in the case of available NPK, the lower levels in untreated plots were due to the mining of available macronutrients with continuous cropping without fertilization for 3 years. The high content of macronutrients in manure-amended plots might be due to the nutrient supplementation to soil through decomposed organic matter which releases CO_2_. The CO_2_ dissolves in water to form carbonic acids and breaks down the minerals which leads to nutrient release [9]. For P, the organic acids released from the decomposed organic manures accelerated the solubilization of P because of improved microbial activities, and retarded P fixation in soil. Higher K content in the soil was due to the enhanced cation exchange capacity of soil which resulted in increased soil capacity for holding exchangeable K and reducing leaching losses of K in soil [46]. In plants involving NPK, the addition of nutrients was done externally to soil which led to the improved nutrient level in soil. These results corroborated well with previous literature where the use of manures and inorganic fertilizers has enhanced the nutrient levels in soil [47, 48].

Different levels of micronutrients in soil with the organic and inorganic amendments were related to the changes in soil environment i.e., less pH and high content of organic matter in soil. The role of manures is not only limited to the supply of in the soil, but it also helps in enhancing biological as well as chemical reactions which leads to the dissolution of non-available micronutrients in soil [49]. These manures along with RSC also form metal complexes with organic matter which improves the levels of available micronutrients by reducing their adsorption, fixation, as well as precipitation. Soil pH decreased with the application of NPK which in turn increased the available micronutrient level in soil. These results corroborated well with the study conducted by Dhaliwal et al. [50], where a considerable upsurge in DTPA-extractable micronutrients was observed through the integrated use of manures and fertilizers under RWCS.

## 5. Conclusions

The present study involved the assessment of the organic manure along with inorganic fertilizer for yield and soil health of the RWCS The application of different manures in addition to RDF significantly increased the grain and straw yield of rice and wheat as well as system productivity over no manure treatments, highlighting the significance of integrated use of fertilizers. The results also showed that the combined use of chemical fertilizers such as NPK with farm yard manure and poultry manure could also resist the decline in system productivity as compared to the long-term use of chemical fertilizers alone. The macro and micronutrient uptake in both crops also increased significantly with the combined application of manures and RDF. Moreover, soil macro and micronutrient status also improved with the exogenous supply of nutrients through 100% RDF and manures. With the increase in the rate of fertilizer dose, grain and straw yield of rice and wheat increased significantly and the maximum was recorded with the application of 100% RDF irrespective of organic amendments except for RSC which showed significantly lower results as compared to other manures. According to this study, the improvement in soil properties, crop growth and system sustainability is highly influenced by balanced chemical fertilization along with regular organic inputs. Together, the findings indicate that it is feasible to increase and sustain the productivity of the RWCS and overall soil quality by applying RDF along with organic manures (FYM, PM and PrM) which further supports the idea of integrating chemical fertilizers with organic inputs to promote system sustainability over time.
